# Application of machine learning and its effects on the development of a nursing guidance mobile app for sarcopenia

**DOI:** 10.1186/s12912-023-01545-w

**Published:** 2023-10-09

**Authors:** Pei-Hung Liao, Yu-Jie Huang, Chen-Shie Ho, William Chu

**Affiliations:** 1 School of Nursing, National Taipei University of Nursing and Health Sciences, Taipei, Taiwan; 2 Department of Nursing, Cheng Hsin General Hospital, Taipei, Taiwan; 3 Department of Healthcare Administration, Asia Eastern University of Science and Technology, New Taipei City, Taiwan; 4 Department of Orthopedics, Cheng Hsin General Hospital, Taipei, Taiwan

**Keywords:** Artificial intelligence, Nursing, Nursing guidance mobile application, Prediction model, Sarcopenia

## Abstract

**Background:**

Aging leads to changes in the body system, such as sarcopenia. This can result in several health issues, particularly physical and mobility dysfunction. Asian people typically have little awareness of sarcopenia. Thus, this study incorporated nursing instruction into the mobile application design to allow users to easily learn about sarcopenia.

**Objective:**

This study evaluated a model for predicting high-risk populations for sarcopenia in home settings. We further developed a sarcopenia nursing guidance mobile application and assessed the effectiveness of this application in influencing sarcopenia-related knowledge and self-care awareness among participants.

**Methods:**

Using a one-group pretest–posttest design, data were collected from 120 participants at a teaching hospital in northern Taiwan. This study used an artificial intelligence algorithm to evaluate a model for predicting high-risk populations for sarcopenia. We developed and assessed the sarcopenia nursing guidance mobile application using a questionnaire based on the Mobile Application Rating Scale.

**Results:**

The application developed in this study enhanced participants’ sarcopenia-related knowledge and awareness regarding self-care. After the three-month intervention, the knowledge and awareness was effectively increase, total score was from 4.15 ± 2.35 to 6.65 ± 0.85 and were significant for all questionnaire items (p values < 0.05). On average, 96.1% of the participants were satisfied with the mobile app. The artificial intelligence algorithm positively evaluated the home-use model for predicting high-risk sarcopenia groups.

**Conclusions:**

The mobile application of the sarcopenia nursing guidance for public use in home settings may help alleviate sarcopenia symptoms and reduce complications by enhancing individuals’ self-care awareness and ability.

**Trial registration:**

NCT05363033, registered on 02/05/2022.

## Background

Aging causes changes in body composition, including a gradual loss of muscle mass. This loss is estimated to decrease by 1% per year after age 30 and accelerates after age 70 [1]. Sarcopenia is associated with numerous adverse outcomes, such as physical and mobility impairments, frailty, falls, fractures, disability, hospitalization, and even death [2]. The National Health and Nutrition Examination Survey conducted in the United States from 1999 to 2004 showed that the prevalence of sarcopenia in adults aged 60 years and older was 29.9% [3]. The awareness and knowledge of sarcopenia among Chinese people is still lacking to this day. In addition, there is a lack of sufficient knowledge and protective behaviors; other comorbidities usually need to appear before the diagnosis can be confirmed. These comorbidities may include fractures caused by falls or immobility, as well as shortness of breath. Consequently, a considerable number of elderly patients are diagnosed only after experiencing dysfunction [2]. The Asian Working Group on Sarcopenia (AWGS) redefined the measurement method in 2019 and reminded the public of the concept of “possible sarcopenia” while promoting the prevention of sarcopenia in mature countries. One can determine whether they are in a high-risk group through a simple behavior test and take preventive and protective behaviors early [4]. In recent years, most studies have focused on accuracy and early diagnosis and treatment recommendations. However, there has been limited attention to home risk evaluations and care tools. Therefore, this study aims to develop a user-friendly program that provides the public with an APP that will enable individuals to screen themselves early to identify high-risk groups, adopt preventive measures to reduce the onset of the disease, and encourage seeking medical treatment promptly for further diagnosis and treatment.

### Sarcopenia definition

The term “sarcopenia” was coined by Rosenberg [5] to describe muscle mass loss with advancing age [6, 7]. Baumgartner et al. [8], Newman et al. [9], and Chen et al. adopted specific percentile or standard deviation approaches to defining sarcopenia; this was measured in terms of included appendicular muscle mass or skeletal muscle mass of people from Asia from the Asian Working Group for Sarcopenia (AWGS) [4]. The Special Interest Group on cachexia-anorexia in chronic wasting diseases under the European Society for Clinical Nutrition and Metabolism contended that, although characterised by a loss of muscle mass and strength, sarcopenia is not exclusively observed in older persons. In 2011, the International Working Group on Sarcopenia defined sarcopenia based on muscle mass loss and reduced physical performance. Moreover, the Society on Sarcopenia, Cachexia, and Wasting Disorders defined sarcopenia as muscle mass loss accompanied by mobility limitations (e.g., changes in walking speed) [3, 10, 11].

In 2019, the Asian Working Group for Sarcopenia (AWGS) in Older People defined sarcopenia as a syndrome characterised by the progressive, generalised loss of skeletal muscle mass and function (i.e., muscle strength and physical activity). It updated Calculus such as calf circumference (< 34 cm in men and < 33 cm in women), SARC-F (≥ 4), and SARC-CalF (≥ 11) to facilitate earlier identification of people at risk for sarcopenia. [4] According to a study conducted in Japan, a sarcopenia diagnosis requires low muscle mass and either low muscle strength or low physical performance. Research has further suggested dividing sarcopenia into three stages: pre-sarcopenia, sarcopenia, and severe sarcopenia [11].

### Nursing guidance

Nursing guidance provides relevant information to patients or caregivers to address their doubts regarding diseases along with appropriate nursing care to satisfy their needs [12, 13]. Nursing guidance through a mobile application (app) offers advantages over other teaching methods. For example, this teaching method can present detected risk factors and allow users to learn about nutrition and exercise through videos whenever required. A mobile app can also be accessed repeatedly at any time and location, saving personnel costs while generating high learner satisfaction [14]. Exercise training and physical activity interventions are crucial to sarcopenia prevention, treatment, and nursing guidance. Common exercise interventions for sarcopenia include the following [15]: (1) modified push-ups for enhancing muscle strength in the shoulders and elbows; (2) squat strength training for enhancing muscle strength in the gluteal muscles and anterior thighs; and (3) foot pointing exercises, which is a muscle strength training exercise involving the upward pointing of the soles of the feet.

### Mobile apps and the artificial intelligence prediction model

A 2015 survey revealed that approximately 83% of the World Health Organisation’s member states had implemented the use of various applications for patient condition tracking and healthcare support (e.g., reminding patients to take their medications, providing guidance on medication use or healthcare decision-making to patients and caregivers) [16]. Artificial intelligence (AI) has rapidly developed in recent years; it is primarily used to predict diseases through machine learning methods, database processing for mining potentially usable information, and discovering trends, characteristics, and relationships in data [17].

Machine learning refers to the process of model construction through AI. This involves searching for and establishing data related to the application context, constructing a data model based on different theories and methods, and enhancing model accuracy through repeated refinement to achieve result prediction [18]. Currently, there are limited methods for assessing mobile health apps’ usability, functions, and quality. Stoyanov et al.’s [19] Mobile Application Rating Scale (MARS) can evaluate the suitability of mobile health apps for enhancing disease awareness among patients and providing nursing guidance [20]. The present study’s assessment findings can be a reference for future app design and development.

## Methodology

### Aim

This study evaluated a model that predicts high-risk populations for sarcopenia in home settings to develop and assess the effectiveness of a nursing guidance mobile app regarding sarcopenia-related knowledge and self-care behaviour among participants.

### Design

**Fig. 1:**
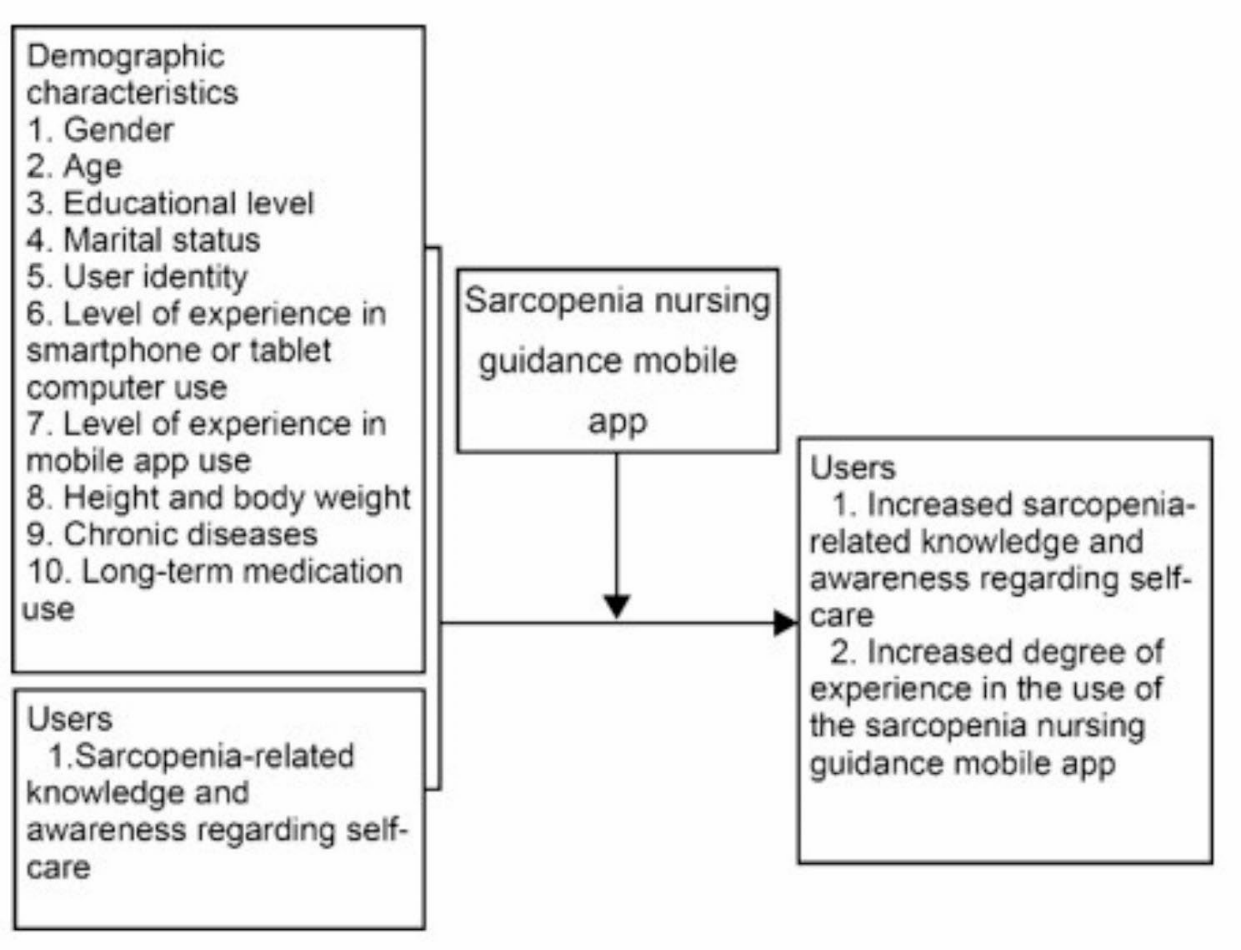
Study Framework

A one-group pretest–posttest design was adopted, and 120 participants were selected through convenience sampling. After establishing and validating a model for predicting high-risk populations for sarcopenia, we developed a mobile app to provide knowledge and nursing guidance to the public. To investigate the effects of the mobile app on sarcopenia-related knowledge and awareness regarding self-care among participants, a self-report survey was conducted before app use and after three months. The mobile app was also assessed using a MARS-based questionnaire after three months (see Fig. 1).

### Participants

The study was conducted in a teaching hospital in Taipei, Taiwan, between 2019 and 2020. Calculations using G-Power 3.1 (assuming power = 0.8, α = 0.05, effect size = 0.35) indicate that at least 108 participants should be included in the study. As such, we adopted the convenient sampling method to recruit 120 participants who visited the hospital regularly. Inclusion criteria were the following: (1) Participants were members of the public aged 20 to 100 years old; (2) They were conscious of or diagnosed by a physician with symptoms of sarcopenia; (3) They had experience using mobile devices or smartphones. Exclusion criteria were the following: (1) Upon diagnosis by a physician, there are no symptoms of sarcopenia; (2) Inability to use a mobile device or smartphone.

### Patient and public involvement

Although patients previously diagnosed with sarcopenia and their families were not involved in designing the research questions or the outcome measures, they were intimately involved in the design and implementation of the intervention. Patients and their families also improved their health knowledge and baseline information’s physiological conditions, which helped motivate home care during and beyond the study.

### Data collection

The study was conducted over one year, from June 17, 2019, to June 17, 2020. Potential participants received adequate information concerning the study in outpatient settings and indicated their intention to participate. Next, participants sequentially completed the informed consent form, demographic questionnaire, and sarcopenia-related knowledge and self-care awareness questionnaire. Upon completing the pretest questionnaires, participants used the sarcopenia nursing guidance mobile app as an intervention measure. After three months of mobile app use, a posttest survey questionnaire was conducted regarding sarcopenia-related knowledge and the participants’ satisfaction with the app. According to the literature, the mobile nursing teaching method can improve changes in knowledge and behaviour approximately the second month after an intervention; the improvement of skills can be observed during the fourth to the sixth month [21, 22]. Therefore, this study took three months to observe the changes in the individual cases after the mobile nursing intervention was implemented.

The researchers collected the questionnaire data for all mobile app users who satisfied the inclusion criteria to maintain consistency. Researchers completed questionnaires for participants who could not complete the questionnaire themselves. Researchers provided assistance and instructions to users regarding using the mobile app. Completed questionnaires were collected by the researchers and appropriately stored in accordance with the principles of confidentiality. During the pilot study stage, participants spent 15–20 min completing the questionnaire.

### Research tools

#### Demographic questionnaire

Demographic data regarding gender, age, educational level, marital status, user identity, primary mobile device use, level of experience with mobile apps, height, body weight, chronic diseases, and medications used were collected. Calf circumference for the lower limb, hand grip strength, and mobility (walking speed) was measured using a simple measuring device (e.g., tape and electronic hand dynamometer). This was operated by the participant or a family member. According to the Ministry of Health and Welfare of Taiwan, calf circumference can be measured at home using ordinary tape oneself or with the help of a family member by adhering to the following steps: (1) Cover the calf with clothes, sit up with both soles of the feet on the ground, and keep the calves at 90 degrees from the thighs; (2) Wrap tape around the calf firmly against the skin without pressing it, and keep the calf at ground level; (3) Measure the widest part of the calf. Additionally, walking speed was observed by patients who recorded individual data after walking about 200–400 m or climbing one or two stairs at normal speed.

#### Sarcopenia-related knowledge and the self-care awareness questionnaire

This study’s questionnaire to assess sarcopenia-related knowledge and self-care awareness was developed based on the relevant literature [4, 11, 23]. It comprised eight items, with Item 1 querying respondents about their sources of sarcopenia-related knowledge. Items 2–8 queried their sarcopenia-related knowledge. Respondents were required to answer “yes” or “no” to each item by selecting the appropriate checkboxes in the questionnaire. According to the Asian Working Group for Sarcopenia (2019) and Kitamura et al. [11] sarcopenia in Asian people can be measured at home according to specific standards. For example, hand grip strength can be replaced by the results of dryness and wetness by wringing towels and handling simple items. In contrast, lower limb muscle strength can be measured by the number of stairs going up and down or whether assistive devices are needed when walking.

The knowledge-related questions were primarily based on the literature and formulated jointly by inviting three experts from relevant fields. The questions are as follows:

**Table:** 

1. I know sarcopenia is a decrease in muscle mass.
2. I know that a simple test method for sarcopenia can be conducted by circling both hands’ thumbs and index fingers around the calf.
3. I know that people are in a high-risk group if they could wring towels and unscrew cans easily in the past but now find it difficult.
4. I know that people are in a high-risk group if it becomes difficult to get up from their seat, such as needing the support of the armrest to get up; it is challenging to climb up 10 stairs, such as taking a break after a few steps.
5. I know that people are in a high-risk group if they lose weight by 5% in 6 months without weight-loss intention. For example, elderly individuals who originally weighed 60 kg but lost 3 kg within six months without any reason will be in a high-risk group.
6. I know people are in a high-risk group if they cannot stand up from a chair without armrests.
7. I know that people are in a high-risk group if it takes them more than 4 min to walk around half the distance of the playground.

In the questionnaire’s final question, users could provide an open-ended response to present their subjective feelings about using this app (such as interface design, content, or other relevant suggestions). Three experts (nursing, medicine, and rehabilitation) were invited to evaluate the appropriateness and clarity of the questionnaire content, and the items were revised based on the evaluation results. The total content validity index (CVI) and Cronbach’s α (internal consistency) of the questionnaire were 0.90 and > 0.90, respectively.

#### Sarcopenia nursing guidance mobile app rating scale

This study used an eight-item questionnaire based on the MARS [20] to rate the mobile app user experience on the MARS’ five quality dimensions: functionality, information quality, aesthetics, subjective quality, and engagement. Respondents indicated their level of agreement with each item on a five-point scale (1 = strongly disagree, 2 = disagree, 3 = neutral, 4 = agree, 5 = strongly agree). This questionnaire could be completed on paper or online through the cloud.

Items 1 “The sarcopenia nursing guidance mobile app can provide me with the knowledge that I require,” 6 “The sarcopenia nursing guidance mobile app can satisfy my current needs,” 7 “I feel that the sarcopenia nursing guidance mobile app is helpful,” and 8 “I will recommend the sarcopenia nursing guidance mobile app to my family and friends,” were related to the functionality dimension. Meanwhile, Items 2 “The sarcopenia nursing guidance mobile app can provide me with relevant exercise methods,” 3 “The sarcopenia nursing guidance mobile app has an easily operable interface and clearly labelled buttons,” 4 “After using the sarcopenia nursing guidance mobile app, my attitude toward appropriate exercising has improved,” and 5 “After using the sarcopenia nursing guidance mobile app, I am reminded of the important points to note when engaging in exercise,” were related to the subjective quality dimension.

We developed the questionnaire content after consulting with researchers and experts in relevant fields. Three experts (a healthcare information specialist, an engineering professor, and a doctor) evaluated the questionnaire content’s appropriateness and clarity. The questionnaire’s total CVI and Cronbach’s α (internal consistency) were 0.90 and > 0.90, respectively.

The cloud-based questionnaire design involved the creation of data collection fields using the prediction model established through machine learning. This enabled the repeated validation of the model accuracy through the continual addition of data.

#### The sarcopenia nursing guidance mobile app

This study developed a sarcopenia mobile app to address issues regarding sarcopenia-related information and nursing guidance identified in the literature [24]. Users could perform the following actions using the mobile app: (1) Gain sarcopenia-related knowledge by accessing the link “Learn more about sarcopenia”; (2) Self-assessment at home (measuring calf circumference, hand grip strength, and walking speed); (3) Watch videos related to exercise and nutrition in sarcopenia; (4) Test user memory regarding sarcopenia-related knowledge through a knowledge questionnaire.

The mobile app, compatible with Android and iOS platforms, was developed using Objective-C and Swift programming.

### Ethical considerations

This study was reviewed by the Institutional Review Board (IRB number: 108 A-06) of the Cheng Hsin General Hospital. All participants were required to read the pre-participation instructions.

### Data analysis

IBM SPSS Windows version 25.0 and IBM Modeller version 18.0 were used for data analysis. Clinical data were analysed with paired t-tests, chi-square, and odds ratios.

#### Establishing and evaluating a sarcopenia risk prediction model through machine learning

The random forest algorithm in the IBM SPSS Modeller was employed to classify sarcopenia risk and validate the prediction model. Random forest operates by constructing many decision tree classifiers using different characteristics, attributes, and data combinations, which are subsequently implemented on randomly allocated training data. Each decision tree classifier in a random forest performs classification and prediction from different data aspects to enhance classification accuracy. Since the Random Forest algorithm is a machine learning algorithm, there is no fixed requirement for calculating the number of samples. Each independent variable requires at least five samples based on the general approximate calculation. Therefore, 120 individuals were recruited to participate in this study, which could be used to build a small prediction model [23].

#### Analysis of web traffic

To determine if and how often users browsed the nursing guidance-related webpages provided by the sarcopenia nursing guidance mobile app and infer its guidance effectiveness, web traffic data were collected using Google Analytics for data organisation and analysis [6]. These results can serve as a reference for formulating website management and developing analytical strategies.

#### Validity and reliability/rigour

Before the study began, a group of experts, including informatics specialists, nurses, and rehabilitation physicians, evaluated and verified the research tools’ effectiveness. The app can provide knowledge regarding sarcopenia and daily care skills and record and analyse whether it is a high-risk group.

## Results

### Demographic variables and correlations

The participants’ average age was 55.12 ± 16.37 years; they were primarily women (69.6%), university graduates (53.6%), married individuals (83.9%), and patients (85.7%). Most participants mainly used smartphones (62.5%), while 32.1% used smartphones and computers. Most participants used mobile apps frequently (39.3%), while 26.8% had no experience using mobile apps. Furthermore, 42% of the participants had chronic diseases, the most common being hypertension (25.9%), followed by diabetes mellitus (10.7%). Additionally, 40.2% of participants used medications long-term, with antihypertensives being the most common (23.2%), followed by hypoglycemic agents (8%) (see Table 1).

The relationship between age and educational level and the level of mobile app experience was determined using Pearson’s correlation coefficient. Age was negatively correlated with the experience level in mobile app use (p = 0.004). Educational level was positively correlated with the level of experience in mobile app use (p < 0.001) (see Table 2).

**Table 1:** Demographic Variables (N = 120)

Categorical variable	Mean ± SD	Number of people (%)
**Age (years old)**	55.12 ± 16.17	
**Gender**		
female		82(69.6%)
male		38(30.4%)
**Educational level**		
Below elementary school		13(9.8%)
Junior high School		11(8.0%)
General and vocational high school Junior college/university or above		34(28.6%) 62(53.6%)
**Marital status**		
Married		96(83.9%)
Unmarried		19(14.3%)
Widowed		5(1.8%)
**Use of main mobile device**		
Intelligent mobile phone		72(62.5%)
Tablet PC		8(5.4%)
Both		40(32.1%)
**Experience of application program**		
Unused		32(26.8%)
Occasionally used		40(33.9%)
Often used		48(39.3%)
**Chronic disease**		
Yes		51(42%)
No		69(58%)
**Long-term medication use**		
Yes		49(40.2%)
No		71(59.8%)

**Table 2:** Correlation Analysis of the Demographic Variables (N = 120)

		Age/P-value	Educational level/P-value	Level of experience in mobile app use/P-value
**Age**	Correlation coefficient, r	1	-0.219*/0.020	-0.4273**/0.004
**Educational level**	Correlation coefficient, r	-0.2.19*/0.020	1	0.428**/0.001
**Level of experience in mobile app use**	Correlation coefficient, r	-0.273**/0.004	-0.428**/0.001	1

*P < 0.05; **P < 0.01

Participants’ average calf circumference was 34.88 ± 4.62 cm, with calf circumference being normal in most of the participants (77.7%) and smaller than normal (< 34 cm in men and < 32 cm in women) in 25 participants (22.3%). The average muscle strength (hand grip strength) was 23.88 ± 10.25 kg, with the values being normal in most participants (68.8%) and lower than normal (< 26 kg in men and < 18 kg in women) in 35 participants (31.3%). The average mobility of the participants was 1.01 ± 0.23 m/s, with mobility being lower than normal in 18 participants (16.1%) and normal in the remaining participants (83.9%) [6].

### Estimated risk of sarcopenia

This study collected data on the presence/absence of chronic diseases, types of chronic diseases, and long-term medications used. The results indicate the presence of 10 types of chronic diseases and 10 types of long-term medications. We determined the associations of each chronic disease and medication type with the three screening indicators for sarcopenia (calf circumference, muscle strength [hand grip strength], and mobility). We used a chi-squared test to evaluate the relationships with sarcopenia, and the risk of sarcopenia was estimated accordingly. Diabetes mellitus was significantly associated with smaller-than-normal calf circumference, lower-than-normal muscle strength (hand grip strength), and mobility.

### Estimated risk of smaller-than-normal calf circumference

Among the various chronic diseases and long-term medications used, diabetes mellitus was associated with a higher risk of smaller-than-normal calf circumference (p = 0.005, odds ratio [OR] = 6.378). The long-term use of hypoglycemic agents also significantly affected the risk of a smaller-than-normal calf circumference (p = 0.025, OR = 5.188).

### Estimated risk of lower-than-normal muscle strength (hand grip strength)

Among the 10 types of chronic diseases, diabetes mellitus was associated with a higher risk of lower-than-normal hand grip strength (p = 0.009, OR = 5.407). Hypertension also significantly affected the risk of lower-than-normal hand grip strength (p = 0.006, OR = 3.375) (see Table 3).

Among the 10 types of long-term medications, antihypertensives, and hypoglycemic agents, they significantly influenced the risk of lower-than-normal muscle strength, as shown in Table 4.

### Estimated risk of lower-than-normal mobility

Among the various chronic diseases and long-term medications used, diabetes mellitus significantly influenced the risk of lower-than-normal mobility (p = 0.024, OR = 4.780). The long-term use of hypoglycemic agents also significantly affected the risk of lower-than-normal mobility (p = 0.036, OR = 5.086), as shown in Table 4.

**Table 3:** Risk of Smaller-Than-Normal Calf Circumference and Muscle Strength Associated with Chronic Disease in the Participants

	Calf circumference				Muscle Strength		
	***X*** ^***2***^	**Odds ratio**	**95%** **confidence interval**	***X*** ^***2***^		**Odds ratio**	**95%** **confidence interval**
**Chronic disease**							
**Hypertension**	0.292	1.479	0.559–3.914	0.006*		3.375	1.393–8.179
**Glaucoma**	0.777	0.989	0.966–1.011	0.687		0.987	0.962–1.013
**Liver disease**	0.398	3.583	0.216–59.429	0.096		1.061	0.978–1.151
**Sciatica**	0.777	0.989	0.966–1.011	0.312		1.029	0.973–1.090
**Diabetes mellitus**	0.005*	6.378	1.816–22.393	0.009*		5.407	1.505–19.426
**Hyperlipidemia**	0.471	1.491	0.271–8.210	0.365		1.766	0.373–8.361
**Chronic asthma**	0.223	1.042	0.962–1.128	0.312		1.029	0.973–1.090
**Rheumatoid arthritis**	0.223	1.042	0.962–1.128	0.312		1.029	0.973–1.090
**Gastroesophageal reflux disease**	0.223	1.042	0.962–1.128	0.687		0.987	0.962–1.013
**Cardiovascular disease**	0.398	3.583	0.216–59.429	0.529		2.235	0.136–36.801

*P < 0.05

**Table 4:** Risk of Smaller-Than-Normal Calf Circumference and Mobility Associated with Long-Term Medication Use

	Calf circumference					Mobility			
	***X*** ^***2***^	**Odds ratio**	**95% confidence interval**	***X*** ^***2***^	**Odds ratio**			**95% confidence interval**	
**Long-term medication use**									
**Cardiovascular medications**	0.535	1.771	0.154–20.375	0.412	2.706			0.232–31.532	
**Antihypertensives**	0.001*	4.500	0.672–4.843	0.207	1.850			0.617–5.543	
**Liver disease medications**	0.777	0.989	0.966–1.011	0.839	0.989			0.969–1.010	
**Stomach medications**	0.535	1.771	0.154–20.375	0.588	0.968			0.933–1.004	
**Hypoglycemic agents**	0.025*	5.188	1.276–21.089	0.036*	5.086			1.217–21.261	
**Hormone medications**	0.535	1.771	0.154–20.375	0.067	11.625			0.995–135.847	
**Hypolipidemic agents**	0.310	2.435	0.384–15.449	0.591	1.324			0.139–12.580	
**Hypnotics**	0.223	1.042	0.962–1.128	0.161	1.059			0.947–1.184	
**Analgesics**	0.223	1.042	0.962–1.128	0.161	1.059			0.947–1.184	
**Traditional Chinese medications**	0.465	0.966	0.928–1.005	0.588	0.968			0.933–1.004	

*P < 0.05

### Prediction model for the risk of sarcopenia

Data from the participants with a smaller-than-normal calf circumference, lower-than-normal muscle strength (hand grip strength), and lower-than-normal mobility were compiled into a database. We used the random forest algorithm to establish the following classification and prediction models for assessing sarcopenia risk: (1) Target variables included smaller-than-normal calf circumference, lower-than-normal muscle strength (hand grip strength), and lower-than-normal mobility. (2) Independent variables included age, gender, body mass index, hypertension, glaucoma, liver disease, sciatica, diabetes mellitus, hyperlipidemia, asthma, rheumatoid arthritis, gastroesophageal reflux disease, cardiovascular disease, cardiovascular medications, antihypertensives, liver disease medications, stomach medications, hypoglycemic agents, hormone medications, hypolipidemic agents, hypnotics, analgesics, and traditional Chinese medications. The model’s accuracy rates for predicting smaller-than-normal calf circumference, lower-than-normal muscle strength (hand grip strength), and lower-than-normal mobility were 73%, 66.7%, and 75.7%, respectively.

### Pretest–posttest comparison of sarcopenia-related knowledge and self-care awareness

Participants’ sarcopenia-related knowledge and self-care awareness questionnaire scores before and after using the mobile app were compared using a one-sample, one-tailed paired t-test. Before the intervention, the mean total score was 4.15 ± 2.35. Item 1, “I am aware that sarcopenia refers to a decrease in muscle mass,” had the highest score among the questionnaire items. In contrast, Item 4, “I am aware that individuals who experience difficulties rising from a chair, such as those who need to use the armrests of the chair to stand, and individuals who experience difficulties climbing 10 steps of a flight of stairs, such as those who need to rest after every two or three steps, are high-risk populations for sarcopenia,” had the lowest score.

After the three-month intervention, the mean total score was 6.65 ± 0.85. Participants obtained the highest and lowest scores for Item 1 (described above) and Item 7, “I am aware that individuals who take more than four minutes to complete the 400 m walk test are a high-risk population for sarcopenia,” respectively. The mean total score of the posttest increased by 2.5 points compared to the pretest. The differences between the pre- and post-intervention scores were significant for all questionnaire items (p values < 0.05). This finding indicates that the intervention significantly improved sarcopenia-related knowledge and awareness regarding self-care, as shown in Table 5.

**Table 5:** Sarcopenia-related knowledge and awareness regarding self-care before and after the intervention (the sarcopenia nursing guidance mobile app)

Effectiveness indicator	Before intervention (N = 120)	After intervention (N = 120)	t	P
	Mean (SD)	Mean (SD)		
**Disease-related knowledge and awareness regarding self-care**				
**Item 1**	0.95(0.23)	1.00(0.00)	-2.51	0.014*
**Item 2**	0.50(0.50)	0.96(0.19)	-9.47	0.001*
**Item 3**	0.70(0.46)	0.99(0.94)	-6.53	0.001*
**Item 4**	0.41(0.49)	0.96(0.21)	-11.52	0.001*
**Item 5**	0.58(0.50)	0.98(0.13)	-8.33	0.001*
**Item 6**	0.58(0.50)	0.94(0.24)	-7.32	0.001*
**Item 7**	0.46(0.50)	0.82(0.39)	-6.15	0.001*
**Mean total score**	4.15(2.35)	6.65(0.85)	-11.16	0.001*

*P < 0.05; SD: standard deviation

### Degree of satisfaction with the app

Results for the sarcopenia nursing guidance mobile app user experience questionnaire indicated a mean total score of 38.43 ± 3.5 out of a maximum possible score of 40. When the individual item scores were classified into “dissatisfied” (1–3 points) and “satisfied” (4–5 points), we found that, on average, 96.1% of the participants were satisfied with the mobile app. Item 2 showed the highest degree of satisfaction (109 satisfied participants, 97.3% satisfaction). Alternately, Item 5 had the lowest degree of satisfaction (105 satisfied participants, 93.8% satisfaction).

Regarding the app’s functionality and subjective quality dimensions, the mean total scores were 19.16 ± 1.838 and 19.15 ± 19.13, respectively, out of a maximum possible score of 20 points for each dimension.

### Post-test qualitative user feedback

We analysed the participants’ open-ended responses on the self-care awareness questionnaire to understand their experiences, feelings, and thoughts regarding their use of the sarcopenia nursing guidance mobile app. Text mining yielded 80 text datasets to analyse keywords in the responses. The results indicate that the highest-frequency terms from the user feedback during the posttest were “simple,” “convenient,” and “easy to use.”

### Web traffic and effectiveness

Google Analytics is a web traffic statistics service provided by Google that provides a Software Development Kit. This is a collection of development tools used by software engineers to create application software for specific software suites and frameworks, hardware platforms, and operating systems. Using Google Analytics for mobile apps allows for the collection and use of information from iOS and Android for tracking each webpage’s click rate and viewing time. According to the data collected, organised, and analysed using Google Analytics on web traffic for the nursing guidance webpages, the users mainly watched the videos regarding rehabilitation and nutrition during the first two months. However, the number of views of the instruction videos for the Yin-Yang conjugation technique and shaking turbidity discharge technique continued to increase with the duration of mobile app use.

## Discussion

### Demographic data

In this study, age was negatively correlated with experience level in mobile app use. Our findings align with previous research that indicates the perceived usefulness of mobile apps, which positively influences the behavioural intention of users from different age groups and identities and with varying levels of information literacy [20, 25].

This study’s findings are based primarily on the experience of participants who are female, college graduates, and married individuals, which may affect the inferences to other groups. This will be discussed in the research limitations section of this paper. At the same time, the results of the present study are similar to those of Batsis, J.A.; Women also have osteoporosis due to the influence of menopausal hormones, which makes them a relatively high-risk group for sarcopenia. The final case collection conditions will be adjusted based on gender in future research. [26].

### Posttest changes in sarcopenia-related knowledge and awareness regarding self-care

The literature on sarcopenia has typically focused on epidemiological surveys, developing health promotion programmes for sarcopenia patients, and nutrition and exercise. Limited studies have investigated the effects of mobile apps on increasing sarcopenia-related knowledge and awareness regarding self-care [27]. In this study, the mobile app significantly improved the participants’ sarcopenia-related knowledge and understanding of self-care, consistent with previous findings [28, 29].

Based on the literature, the Rapid Geriatric Assessment (RGA) using Mobile App is a fast and feasible tool that can identify geriatric syndromes and assist management in primary nursing. Healthcare providers can use it in hospitals or at home to identify high-risk groups [30]. The results of the study are similar to those of this study. Individual cases are expected to manage their health status at any time and moderately adjust their self-care behavior in daily nursing. Additionally, another study mentioned that several tools for early detection of geriatric syndromes had been developed over recent years. These are available to use at home, such as EasyCare, Gérontopôle Frailty Screening Tool, Rapid Geriatric Assessment, and Kihon Checklist [31]. The present study primarily intends to identify home and invasive high-risk risk factors for public use.

### Degree of satisfaction with the app

We used a sarcopenia nursing guidance mobile app instead of conventional health education or face-to-face teaching to enhance sarcopenia-related knowledge and self-care awareness. Assessment of the participants’ satisfaction with the mobile app after intervention revealed a mean total score of 38.43 ± 3.5 over 40, with 96.1% reporting satisfaction with the mobile app. In the healthcare industry, healthcare professionals typically disseminate disease-related knowledge and nursing care guidance through health education to enhance the knowledge and self-care awareness of the general public. However, health education is typically conducted face-to-face. It involves providing a considerable amount of information quickly, making it challenging for the public or patients to assimilate this information [32] entirely. This issue may be addressed by making health information readily accessible through mobile devices and apps, which can significantly benefit the public and patients by allowing more time to acquire disease-related knowledge. Other studies have also reported that interventions using information-providing mobile apps enabled the general public’s rapid acquisition of knowledge; these apps provided effective and timely health information and achieved high levels of satisfaction among users [23, 33, 34], which is consistent with our findings.

### Limitations

During the evaluation of the home-use model, we could not measure participants’ muscle mass using dual-energy x-ray absorptiometry or bioelectrical impedance analysis. Therefore, data on chronic diseases and long-term medication use were collected instead for the correlation analysis and risk estimation of smaller-than-normal calf circumference, lower-than-normal muscle strength (hand grip strength), and lower-than-normal mobility. This study’s important interference factors included the urgent need to use mobile health devices during the COVID-2019 period, which may have affected the data collected from certain populations. Further in-depth exploration will be conducted in future studies.

## Conclusion

This sarcopenia nursing guidance mobile app can provide an approach to nursing for high-risk group cases, which has significantly improved the understanding of sarcopenia and self-care awareness. Furthermore, diabetes significantly impacts the risk of having less than normal calf circumference, muscle strength (hand grip strength), and mobility. Taking antihypertensive drugs can also affect the occurrence of sarcopenia. These findings should be integrated into nursing guidance and are helpful in developing self-assessment tools forsarcopenia in the home environment.

## Data Availability

The data presented in this study are openly available in FairShare repository, https://www.rd-alliance.org/node/58743/file-repository.

## Abbreviations

APP: Application
AI: Artificial intelligence
AWGS: Asian Working Group for Sarcopenia
MARS: Mobile Application Rating Scale
CVI: Content validity index
OR: Odds ratio
